# The systemDrive: a Multisite, Multiregion Microdrive with Independent Drive Axis Angling for Chronic Multimodal Systems Neuroscience Recordings in Freely Behaving Animals

**DOI:** 10.1523/ENEURO.0261-18.2018

**Published:** 2019-01-07

**Authors:** Myles W. Billard, Fatemeh Bahari, John Kimbugwe, Kevin D. Alloway, Bruce J. Gluckman

**Affiliations:** 1 Department of Engineering Science and Mechanics, Penn State University, University Park, Pennsylvania 16802; 2 Center for Neural Engineering, Penn State University, University Park, Pennsylvania 16802; 3 Department of Neural and Behavioral Sciences, Penn State University, University Park, Pennsylvania 16802; 4 Department of Neurosurgery, Penn State University, University Park, Pennsylvania 16802

**Keywords:** chronic, electrophysiology, freely behaving, microdrive, systems neuroscience, tetrode

## Abstract

A multielectrode system that can address widely separated targets at multiple sites across multiple brain regions with independent implant angling is needed to investigate neural function and signaling in systems and circuits of small animals. Here, we present the systemDrive, a novel multisite, multiregion microdrive that is capable of moving microwire electrode bundles into targets along independent and nonparallel drive trajectories. Our design decouples the stereotaxic surgical placement of individual guide cannulas for each trajectory from the placement of a flexible drive structure. This separation enables placement of many microwire multitrodes along widely spaced and independent drive axes with user-set electrode trajectories and depths from a single microdrive body, and achieves stereotaxic precision with each. The system leverages tight tube–cannula tolerances and geometric constraints on flexible drive axes to ensure concentric alignment of electrode bundles within guide cannulas. Additionally, the headmount and microdrive both have an open-center design to allow for the placement of additional sensing modalities. This design is the first, in the context of small rodent chronic research, to provide the capability to finely position microwires through multiple widely distributed cell groups, each with stereotaxic precision, along arbitrary and nonparallel trajectories that are not restricted to emanate from a single source. We demonstrate the use of the systemDrive in male Long–Evans rats to observe simultaneous single-unit and multiunit activity from multiple widely separated sleep–wake regulatory brainstem cell groups, along with cortical and hippocampal activity, during free behavior over multiple many-day continuous recording periods.

## Visual Abstract

**Figure:**
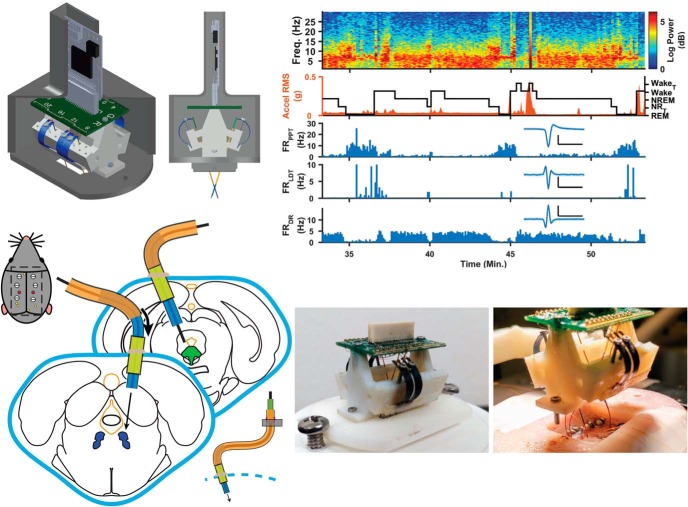

## Significance Statement

The novel design of the systemDrive overcomes current microdrive limitations by providing the ability to finely position microwires through multiple widely distributed cell groups, each with stereotaxic precision, along arbitrary and nonparallel trajectories that are not restricted to emanate from a single source. This allows for the targeting of a larger set of brain regions, including deep brainstem areas because the electrodes do not emerge from a single area, initial driving points can be placed at set distances from targets and electrodes can be placed along trajectories designed to minimize collateral damage to sensitive brain structures. This technology enables easier simultaneous monitoring and analysis of neural activity in multiple widely spaced brain regions that comprise a fully interconnected neural circuit.

## Introduction

There is a substantial interest in elucidating the circuit-wide interactions that mediate specific behaviors and neural functions (Carandini, 2012; Bassett and Sporns, 2017). Imaging techniques, such as fMRI (van den Heuvel and Hulshoff Pol, 2010), fiber tractography (Shi and Toga, 2017), and calcium imaging (Kim et al., 2016) have provided insights into the connectomes that underlie neural circuits (Swanson and Lichtman, 2016). Still, there is a gap in the understanding of real-time neurophysiological signaling and dynamics for most neural networks. This is largely due to the lack of tools designed for collecting electrophysiological data from multiple interconnected brain regions during free behavior.

**Figure 1.**
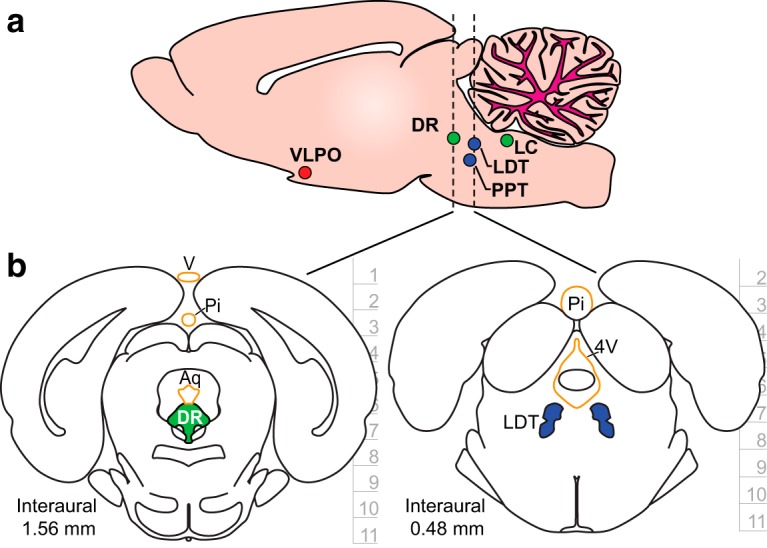
Targeting the sleep–wake regulatory network. ***a***, A sagittal brain section with structures implicated in sleep–wake regulation. The structures are distributed across multiple regions of brainstem. Color codes represent the postulated states of vigilance during which specific cell populations within each structure are predominantly active: red is non-REM (NREM) sleep; blue is REM sleep; and green is wake. Brain region names and acronyms are as follows: ventrolateral pre-optic nucleus (VLPO), laterodorsal tegmental nucleus (LDT), pedunculopontine tegmental nucleus (PPT), dorsal raphe (DR), and locus coeruleus (LC). ***b***, Coronal brain sections that highlight SWRS populations DR and LDT underneath sensitive anatomic structures highlighted in orange. These dorsal structures should not be damaged during chronic recording studies, and so safe access to the targets requires independent and angled electrode trajectories. Labels are as follows: vein (v), pineal gland (Pi), cerebral aqueduct (Aq), and fourth ventricle (4V). Depth information is in millimeters with horizontal tics representing 1 mm. Coronal sections were adapted with permission from Paxinos and Watson (2007, their Figs. 95 and 104; copyright Elsevier).

Many networks that control fundamental physiologic behaviors involve cell populations that are distributed across different regions of brain. A primary example is the sleep–wake regulatory system (SWRS). Shown in Figure 1*A* are several brain structures with cell groups thought to be responsible for controlling the different behavioral states of sleep (Datta and MacLean, 2007; Saper et al., 2010; Booth and Diniz Behn, 2014). These structures, ventrolateral preoptic nucleus (VLPO), laterodorsal tegmental nucleus (LDT), pedunculopontine tegmental nucleus (PPT), dorsal raphe (DR), and locus ceruleus (LC), reside in separated brainstem regions, and there is no single linear trajectory along which an implant can access all these targets. Some of the cell groups are also positioned underneath sensitive anatomical structures, such as ventricles and veins, that should not be damaged by implants for the chronic survival of recording subjects (Fig. 1*B*). These are some of the challenges that make it difficult to hit all of these deep areas simultaneously. A multielectrode system that can target specific areas across multiple brain regions and can independently angle implants is needed to record from such widely distributed neural circuits.

Multielectrode static implants and microdrives are attractive tools for chronically recording multiple brain targets in a behaving animal. Although static electrode implants are used for large-scale multisite recordings (Nicolelis et al., 2003; Xie et al., 2016), their yields and stability are limited by the accuracy of electrode placement. Additionally, their signal stability can degrade over time from local glial scarring and cell death (Polikov et al., 2005; Griffith and Humphrey, 2006; Vaidya et al., 2015). Microdrives, however, provide the ability to move electrodes to improve the quality of recordings and increase sampling of chronically recorded neurons (Wilson et al., 2003; Jackson and Fetz, 2007; Voigts et al., 2013). This makes them a preferred tool for chronic recording studies.

One major downside of current microdrives is the lack of designs that accommodate multiple nonparallel electrode trajectories. Most microdrives are single-site devices that use a high number of electrodes that funnel to a central convergence point for motion along a single axis. While this type of design is successful for place cell studies (Gothard et al., 1996; Battaglia et al., 2009) and other regional observations where targets are aligned along the same axis (Dobbins et al., 2007; Miyakawa et al., 2012; Voigts et al., 2013; Márton et al., 2016), they are not suitable for multisite, multiregion (MSMR) recordings. A selection of microdrives offer MSMR targeting capabilities (Lansink et al., 2007; Yamamoto and Wilson, 2008; Santos et al., 2012; Dotson et al., 2017; Liang et al., 2017) or have small enough footprints to be used in multiple places on a subject (Headley et al., 2015; Nano-Drive, Cambridge NeuroTech), but these designs are limited by their fixed, parallel implant geometries or cannot be used in small animals. The “Quad-Drive” (Vinck et al., 2016; Bos et al., 2017), a derivative of the Split-Drive from Lansink et al. (2007), achieves nonparallel electrode trajectories via angling of individual exit cannulas. However, its electrode trajectories start from the same cortical position, which limits the ability to design trajectories that avoid sensitive structures.

Here, we present the systemDrive, an MSMR microdrive with independent and arbitrary positioning for multiple electrode axes. The systemDrive uses flexible polyimide tubing within a decoupled guide-and-placement cannula system to create user-defined drive trajectories and implant depths for observation of many widely separated brain targets. The systemDrive supports multimodal electrophysiology through its open-center design, wherein auxiliary sensors can be implanted alongside microwire implants. We have routinely targeted three to five independent nonparallel axes spread through brainstem for recording SWRS structures in rats. To demonstrate that the systemDrive can meet the demands of chronic large-scale electrophysiology in small freely behaving animals, we assessed targeting accuracy, chronic stability, and the relationship between neural activity and behavioral state as scored from cortical and hippocampal field potentials as well as animal motion.

## Materials and Methods

This section is divided into five parts (1) design and preparation of standard microdrive elements, (2) surgical steps for chronic survival surgery, (3) protocols for chronic recordings and electrode-driving sessions, (4) data analysis methods, and (5) histologic protocols.

### Hardware preparation

#### 3D printed materials

All 3D printed devices and assemblies described in this manuscript were designed with AutoCAD (Autodesk).

Microdrive bodies were printed with an Objet260 Connex (Stratasys) printer with Vero PolyJet material. Drive screw holes and flexible drive axis (FDA) holes were cleared using size #71 and/or #64 drill bits in preparation for thread tapping.

The headmount cap and base, and microdrive placement tool, were printed using a uPrint SE (Stratasys) 3D printer.

#### Drive springs

Drive springs were cut from 2 mil (0.002 inches) blue spring steel shim stock (McMaster-Carr) using a water jet cutting process on an Omax JetMachining abrasive waterjet (Omax). The shim stock was temporarily coated with pure white beeswax to prevent corrosion from exposure to the water jet cutting process.

#### Microwire bundle preparation

Microwire bundles (*n* = 4 or *n* = 6 wires/bundle) were constructed using 25.4 µm diameter formvar-insulated nichrome wire (A-M Systems) on a Tetrode Assembly Station (Neuralynx) and followed standard protocols for tetrode fabrication (Nguyen et al., 2009; Chang et al., 2013).

#### Microwire electrodeposition

Custom-printed circuit boards (PCBs) were designed using AutoCAD Eagle (Autodesk) software and manufactured by Advanced Circuits. Microdrive electrode interface boards (EIBs) were fabricated with two 31 mil thick copper layers and a gold-immersion finish.

Electrical connections to microwire electrodes were achieved by riveting insulated leads into vias (16 mil diameter) of an EIB using small gold pins (Neuralynx). A channel-access board connected the EIB to a custom galvanostat/potentiostat circuit board that used Labview (National Instruments) scripts for both microwire electrodeposition and impedance testing. Electrodeposition of gold onto exposed microwire recording sites reduced the impedances of electrodes to 200–350 kΩ. Before electrodeposition, the tips of microwire bundles were cleaned in a 30 ml ultrasonic bath of acetone then rinsed in deionized (DI) water. A solution of 90% DI water and 10% noncyanide gold solution (catalog #5355, Sifco ASC) was used for gold plating. Microwires were connected as working electrodes in a three-electrode configuration with platinum reference electrodes and counterelectrodes inside the gold solution. A current of –2 µA was applied to each working electrode during deposition. After deposition, microwires were rinsed in DI water and isopropyl alcohol, then retracted back into microdrive tubing.

### Surgery

All animal procedures were performed in accordance with the regulations of the Penn State University Animal Care Committee.

#### Chronic survival surgery

Male Long–Evans rats (275–350 × *g*) were anesthetized with injections of ketamine (50 mg/kg, i.m.) and xylazine (10 mg/kg, i.m.), intubated, and maintained under isoflurane anesthesia throughout the surgery. Dexamethasone (5 mg/kg, s.c.) was administered before surgery. Body temperature was maintained with a heating blanket, and saline (∼2 ml/h, s.c.) was administered throughout. Animals were mounted into a digital readout stereotaxic device (Angle One, MyNeurolab).

#### Microdrive placement procedure

Implant details critical to the microdrive design are outlined in the Results (Microdrive placement).

### Recordings

#### Chronic, freely behaving recordings

Chronic and continuous recordings were conducted in freely behaving animals over an average of 5 to 7 d for each recording period. Data were acquired with Intan RHD2116 differential or an RHD2132 referential amplifiers with three-axis accelerometers and an RHD2000 Evaluation System (Intan Tech). Neuronal signals were acquired at 30 kHz and high-pass filtered at 1 Hz, and acceleration measurements were sampled at 7500 Hz. Animals were cabled to 3´ lightweight SPI cables (Intan Tech) attached to low-torque SRA-73540-12 slip-ring commutators (Moog) via a custom interface PCB. Animals were continuously monitored with video in their home cages and given access to food and water *ad libitum*.

#### Electrode driving

Electrode bundles were initially implanted with tips located linearly ≥1000 µm away from their targets to avoid glial scarring of regions of interest due to implant trauma. Pretarget electrode driving sessions were typically performed across five sessions, normally 2–4 d apart, to prevent stressing the animal. For these sessions, the rat was anesthetized with isoflurane. Individual electrode bundles were driven ventrally through the brain by turning independent drive axis screws. Electrode tip distances were estimated *in vivo* by calculating the linear distance traveled, based on the number of screw turns made, from the initial stereotaxic position of the electrodes, as well as from expected neural activity. The maximum driving distance for an electrode bundle in a session was typically between 212 and 318 µm, or ∼1–1.5 turns of the drive screw. Electrodes were advanced larger distances during pretarget driving sessions, but, once electrodes were estimated to be within their targets, their tip positions were adjusted more finely in typically 35–106 µm increments to isolate new neurons. Driving sessions were performed between individual recording periods (typically, 5–7 d/period) after electrodes were estimated to be within their targets.

### Data analysis

All analyses were performed using custom-written MATLAB and Labview scripts.

Accelerometer, electrocorticogram (ECoG), and hippocampal local field potential (LFP) time series were downsampled to 1000 Hz and reformatted into hour-long blocks of binary data. Raw signals were bandpass filtered at 2–100 Hz (accel), 1–55 Hz (ECoG), and 1–125 Hz (LFP). EEG power spectra were computed with overlapping 10 s windows (5 s overlap) for spectrograms and sleep scoring. The root mean square (RMS) of filtered head acceleration was calculated with 5 s sliding windows with 1 s overlap. State of vigilance (SOV) was semiautomatically marked according to methods used in the studies by Sunderam et al. (2007) and Sedigh-Sarvestani et al. (2014) with a Labview script.

Segments of raw brainstem recordings were bandpass filtered consecutively at 250–7.5 kHz. The initial filter states were saved and used recursively to avoid discontinuities. Thresholds were then applied to the data segments to enable the detection of individual units that surpassed 5–7 SDs of the mean of the filtered signal. Single units were then sorted into clusters within custom-written MATLAB scripts adapted from the UltraMegaSort2000 toolbox (Hill et al., 2011).

For the purposes of this work, we investigated the neuronal activity of the target structures as a function of SOV. The single neurons were later marked as being state dependent if their activity significantly increased during a certain SOV. We further validated these neurons by waveform morphology compared with published reports from comparable regions (Gervasoni et al., 2000; Datta and Siwek, 2002; Dahan et al., 2007; Sakai, 2011).

### Histology

Animals were deeply anesthetized with mixture of ketamine (60 mg/kg) and xylazine (18 mg/kg), and then were transcardially perfused sequentially with solutions of saline-heparin, a 4% paraformaldehyde solution, and a 4% paraformaldehyde/10% sucrose solution. After decapitating the rat, the head with the implanted electrodes was placed in a 4% paraformaldehyde/30% sucrose solution for ∼24 h. Brains were then extracted from the bottom of the cranium to minimize potential tissue damage as the brain was separated from the implants. Subsequently, the brain was immersed in a solution of 4% paraformaldehyde/30% sucrose until it sank.

Coronal brain sections were obtained with a freezing microtome at a thickness of 60 µm and were rinsed in 0.1 m PBS. Sections were then either stained with cresyl violet or processed for the presence of NADPH. NADPH staining was performed according to the procedures used in the study by Vincent et al. (1983). Briefly, brain slices were agitated in a solution of 0.1 m PBS, pH 8.2, and 0.35% Triton X-100 (Sigma-Aldrich) for 1 h. Sections were then incubated for ∼2–3 h in Triton X-100 solution containing 1 mg of Nitrotetrazolium Blue Chloride (catalog #N6639, Sigma-Aldrich) and 0.1 mg/ml Nitrotetrazolium Blue Chloride (catalog #N6639, Sigma-Aldrich). At the end of the staining procedure, sections were rinsed in 0.1 m PBS and mounted on gel-dipped glass slides. In both cresyl violet and NADPH staining cases, the mounted sections were dehydrated in an incubator and coverslipped with Cytoseal.

## Results

The results are presented in two parts. First, the novel design of the microdrive and associated surgical techniques are described in detail. Second, we present *in vivo* histology and neurophysiology data that demonstrate the accuracy and reliability of the microdrive. The files needed to build the device described in the article are freely available on-line at https://doi.org/10.18113/S1Q64M.

### The flexible drive axis

**Figure 2.**
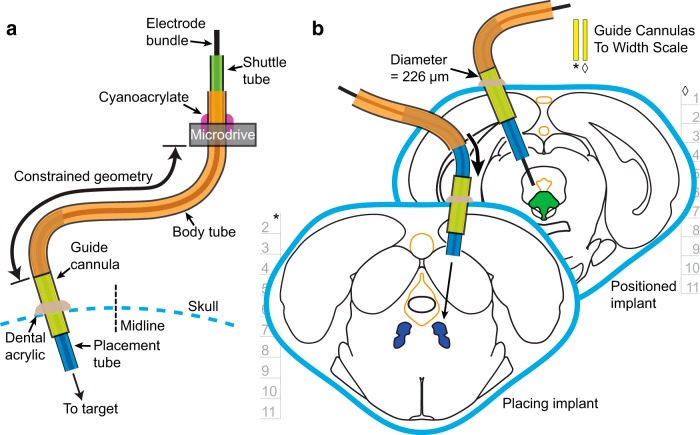
The flexible drive axis. ***a***, Diagram of the FDA. The FDA has a long flexible body tube that is fixed at the top to the microdrive and has a free end with a placement tube. The free implant end is inserted into a skull-fixed guide cannula contralateral to the microdrive fixed point. This creates a second fixed point on the axis. The constrained geometry allows the electrode bundle to move precisely through the tubing and to exit the placement tube at the angle set by the guide cannula. ***b***, Placement of FDAs to multiple targets from multiple implant sites. The independent angling of each FDA provides maximal spatial recording coverage of targets while avoiding sensitive brain structures. During FDA positioning, the placement tube is slid into the guide cannula (diameter, 226 µm). The FDA is positioned once the body tube meets the top of the guide cannula. Depth information is in mm with horizontal tics representing 1 mm. Width-scaled guide cannulas (front section, *; back section, ◊) have been included to better contextualize potential damage to brain from the implant. FDA components on both subpanels are not drawn to scale. Coronal sections were adapted with permission from Paxinos and Watson (2007, their Figs. 95 and 104; copyright Elsevier).

The core innovation of the systemDrive is the flexible drive axis (FDA, Fig. 2*A*). The FDA is a polyimide tube structure with a fixed end on the microdrive body, a shuttle tube, and a free implant end. The free implant end consists of a long body tube and a placement tube (Table 1; polyimide tubing, A-M Systems). To define a complete driving axis, the free implant end is inserted into a skull-fixed guide cannula, which is positioned contralateral to the fixed point on the microdrive. The placement tube slides into the guide cannula until the body tube meets the top of the cannula, which creates a second fixed point on the FDA. The two fixed points—the microdrive and the guide cannula—define a constrained geometry through which the electrode bundles move. This two-point-wide radius curvature prevents the FDA from kinking and minimizes electrode bundle binding.

**Table 1:** FDA tubing parameters

Element name	Tube diameter (mil)	Tube length (mm)
Shuttle tube	6.3	8–10
Body tube	8.9	17–18, 16–17
Placement tube	6.3	11–12, 13–14
Guide cannula	8.9	6–6.5

Tube names with associated diameters and lengths that are used as part of the flexible drive axis. The two sets of values in the tube length cells of the body tube and placement tube are the ranges of lengths used when constructing flexible implants for SWRS targets DR, PPT, LDT, and LC (*R* ∼ 9 mm) and deeper VLPO targets (R ∼ 11 mm).

The guide cannula and the placement tube have a difference *δr* = 28 µm between their respective inner and outer diameters for the tubing stock chosen. This creates concentric alignment in the FDA to ensure that the electrode bundle does not significantly deviate from the user-set trajectory angle of the drive axis. For a given cannula length *l*, the maximal angular deviation of the electrodes will be of order *dθ* ∼≃ *δr* ∕ *l*, with a maximal targeting error at target depth *D* of *Dδr* ∕ *l*. For values of *D* = 7 mm and *l* = 2 mm, this accounts for a maximal targeting error of 98 µm. When fully positioned, the electrodes emerge straight from the ventral end of the FDA at a precise driving angle and distance to target. Shown in Figure 2*B* is a conceptual diagram of the placement and positioning of two independent FDAs.

**Figure 3.**
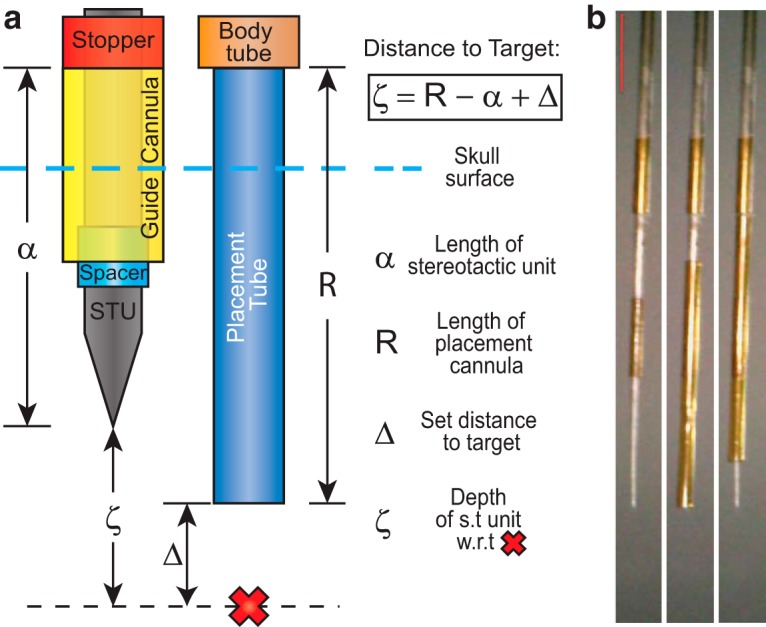
Stereotaxic targeting diagram. Diagram of the FDA stereotaxic targeting method and the stereotaxic unit (STU). ***a***, The ventral end of the FDA can be implanted at a user-defined absolute driving distance Δ away from a target, indicated by the red X, along any arbitrary trajectory. This is achieved by using the STU to carry the guide cannula to the correct coordinates and implant depth. The top of the guide cannula is the same diameter as the body tube (8.9 mil) and thus sets the stopping point of the placement tube (6.3 mil). The difference between the length of the placement tube *R* and the length from the top of the guide cannula to the tip of the STU α is known by subtracting the two premeasured values. Thus, ventral depth of the FDA is guaranteed by implanting the STU a distance ζ from the target, which is calculated using the Distance to Target equation. ***b***, A series of photographs showing the STU without and with a guide cannula attached. The STU is a surgical stylus that contains a spacer tube (6.3 mil) and a stopper tube (8.9 mil). The guide cannula is slid onto the STU until it reaches the stopper tube. During surgery, the STU is implanted, the guide cannula is fixed to the skull, and then the STU is removed, which leaves just the guide cannula in place. Red scale bar, 2 mm.

**Figure 4.**
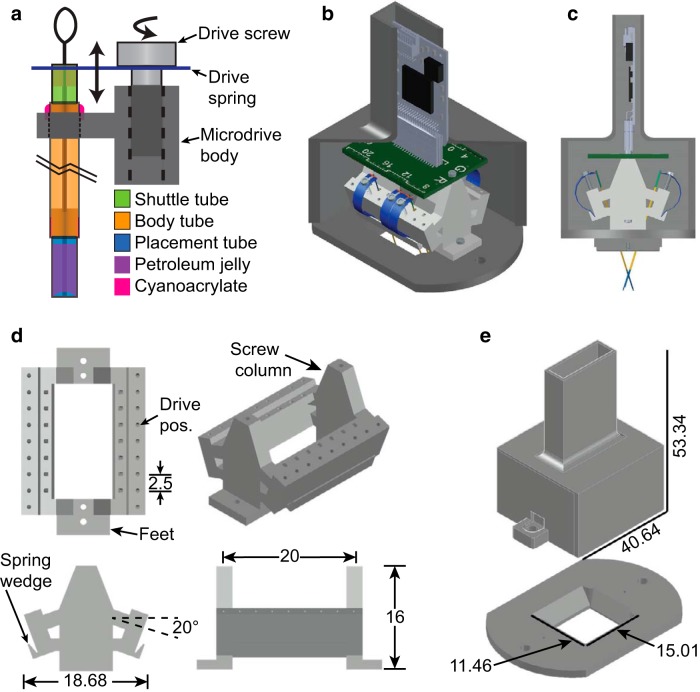
Microdrive system details. ***a***, The FDA is made of a tubing structure that encloses a microwire electrode bundle as it is pushed through the drive axis by a drive spring. The shuttle tube is rigidly attached to a bridge on the head of the drive spring and can move through the body tube. As the drive spring is moved up or down with the turning of a drive screw, the shuttle tube and electrodes move with the spring head. The body tube is fixed to the microdrive body with cyanoacrylate. The body tube is very long, as marked by the black break lines. The placement tube is fixed within the bottom of the body tube. Petroleum jelly space fills the ventral end of the placement tube, except at the electrode tips, to prevent fluids and proteins from entering the drive axis and binding the electrode bundle. ***b***, ***c***, Isometric (***b***) and side (***c***) 3D-rendered computer-aided design (CAD) model views of the complete microdrive system to scale. Elements of the system include the following: the microdrive body (light gray structure), drive springs and screws, FDAs, an electrode interface board (green printed circuit board) with a 36-position Omnetics connector, an Intan RHD2216 amplifier, and a headmount (dark gray base and cap). The cap of the headmount has been sliced to view the elements inside. ***d***, Top, front, side, and isometric 3D-rendered CAD model views of the microdrive body. The four primary features on the microdrive body are: the drive positions, the screw columns, the base feet, and the spring wedges. The two drive position arrays have tap holes for 000-120 threads and are angled at 20° with respect to the level screw columns. The 20° provides clearance for the screwdriver when driving and allows the long flexible implants to naturally extend over midline, as depicted in the side view of ***c***. The high-density design has 14 usable drive positions to allow for the targeting of implant sites along an extended rostrocaudal range. Each individual drive position is 2.5 mm in width and includes a drive axis hole for fixing the drive axis bundles to the microdrive body. The two screw columns have 00-90 tap holes and are spaced apart by 20 mm. The feet of the microdrive have 00-90 through-holes and are where the microdrive body is secured to the headmount base. The 1 mm tall spring wedge is on the outside of the drive position arrays and is angled at 20°. The microdrive body is 20 mm in length, 16 mm in height, and 18.68 mm in width. ***e***, Isometric 3D-rendered CAD model views of the headmount. The headmount is composed of two components: the base and the cap. The base has a 4-40 tap hole for the cap screws and a smaller 0.66 mm hole that gets enlarged and tapped with 00-90 threads for the foot screws that secure the microdrive body to the headmount base. The base has a centered 15.01 × 11.46 mm opening for access to the cranium, which allows the placement of multiple guide cannulas and extrasensor modalities. The opening has sloped side and back walls to provide extra room for the maneuverability of forceps when placing the microdrive onto an animal. The 3D CAD models for the Omnetics Nano Strip connector and Intan RHD2216 amplifier were downloaded from the Omnetics (part A79026-001, http://www.omnetics.com/products/neuro-connectors/nano-strip-connectors) and Intan (http://intantech.com/downloads.html) web sites.

**Figure 5.**
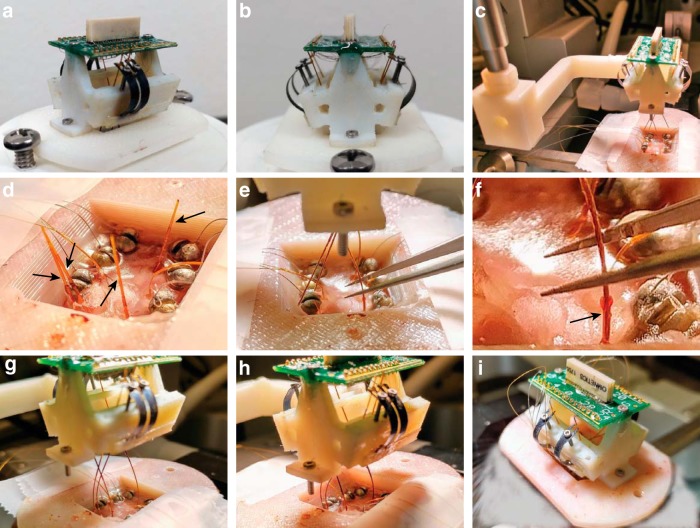
Microdrive placement. ***a***, ***b***, Photographs of a fully constructed microdrive with four drive axes. ***c–i***, Chronological photographs from a microdrive placement procedure with the following four nonparallel, separated drive axis targets: VLPO, DR, PPT, and LDT. ***c***, The microdrive is attached to a custom 3D-printed placement tool, which can be manipulated by the stereotaxic device. ***d***, Petroleum-jelly plugs, highlighted by the four black arrows, are first removed from the skull-fixed guide cannulas. Leads of the accessory electrodes, which include cortical screws and hippocampal depth electrodes, are taped down to the side of the headmount base for later riveting. ***e***, ***f***, The microdrive is lowered with the stereotaxic device, and the placement tubes of each drive axis are inserted into the skull-fixed guide cannulas, one of which is highlighted with a black arrow in ***f***. The placement tubes are guided using customized forceps that have small notches near the tips to grab and manipulate the tubes without crushing them. ***g***, All four placement tubes are engaged with their respective guide cannulas. ***h***, The placement tubes are closer to being in complete position. Once the drive axes are in place, the bottom of the body tube, which is highlighted with black marker, will be seated on top of the guide cannula. ***i***, After all the drive axes are seated, the microdrive is secured to the headmount base with the foot screws and the placement tool is removed. Finally, the cortical screw and depth electrode leads are riveted into the EIB.

The full design of the systemDrive incorporates a range of elements that includes the following: the FDA; the skull-fixed guide cannula and the tool and surgical methodology to implant them, illustrated in Figure 3; the microdrive body, along with its enclosure, depicted in Figure 4; and the protocol for implanting the full drive, shown in a series of snapshots in Figure 5. These elements are discussed in detail in the following sections.

A surgical method was developed alongside the design of the FDA to enable accurate trajectory and depth settings of FDA implants. The method uses standard stereotaxic procedures that define a linear trajectory through a target and a coordinate system along that axis. A craniotomy is made along the target trajectory, and a guide cannula is positioned so that its dorsal end will prevent the FDA from advancing further (Figs. 2*A*, 3*A*). This is achieved by stereotaxically placing the guide cannula using a surgical stylus denoted as the stereotaxic unit (STU; Fig. 3) and then sliding the placement tube of the FDA (Fig. 5*E–H*), which is attached to the microdrive body (Fig. 4*A–C*), through the guide cannula that is fixed to the skull.

Figure 3*A* depicts the geometry of the STU with the guide cannula and lower portion of the FDA, as well as their relationship along the trajectory to the target, and Figure 3*B* includes a series of images of the STU without and with a guide cannula. The STU is composed of a commercially fabricated tungsten electrode (ID UEWMFFSECNNM, FHC), a 6.3 mil tube used as spacer to keep the cannula concentrically aligned with the STU, and a 8.9 mil stopper tube. The distance from the STU tip to the stopper is defined as α. The FDA moves through the guide cannula a distance *R*, such that the body tube portion meets the top of the cannula. These parameters can be measured using a microscope line length tool or a physical scale with at least 100 µm resolution. The values *R*, α, and the electrode driving distance Δ are input into Equation 1 to calculate the distance along the implant axis ζ the STU tip must be from the target coordinates to have the FDA end a distance Δ from the target, as follows:

(1) ζ=R−α+Δ.

The targeting parameters *R* and α must be adjusted depending on the dorsoventral (DV) coordinate of the target. There is flexibility in choosing these values, and the system is not limited by the depth or shallowness of specific targets. For more ventral targets, such as any of the SWRS targets discussed in this article, ζ should be increased to minimize potential damage caused by the STU. To do so, *R* can be made several millimeters larger than α, which can be maintained within a 7–7.5 mm range. For more superficial targets, ζ should be smaller, and an STU with an α value closer to *R* should be used.

At any depth, the top of the guide cannula needs to extend out of the dental cement used to fix the cannula and headmount to the skull and thus must be above the cranial surface. As such, α + ζ > |DV Depth + 0.5mm| must hold true, where DV depth is the anatomic depth of the target and 0.5 mm is an extra safety factor for the application of dental acrylic. At its most shallow, the bottom of the guide cannula needs to be anchored in the bone. In this case, α − |Guide Tube Length| < |DV Depth| can be used as a check. For targeting dorsal cortical layers, the guide cannula and placement tubes need not penetrate the parenchyma, and an STU fabricated without a portion that extends beyond the bottom of the guide cannula can be used.

### Microdrive elements and design

The systemDrive uses recent innovations in driving mechanisms (Voigts et al., 2013) and variations on classic microdrive tubing structures. The system can be subdivided into key elements that, when combined, make the microdrive function. These key elements are shown in Figure 5 and include: the FDA, the driving mechanism, the microdrive body, the electrode interface board, the recording amplifier, and the protective headmount.

Shown in Figure 4*A* is a diagram of the FDA attached to the microdrive and driving mechanism. As with other microdrives, the systemDrive uses a tube-within-tube structure to guide the path of the microwire bundle. A shuttle tube is maneuvered up and down by a drive spring head and carries an electrode bundle through a longer body tube and placement tube. The body tube is fixed to the microdrive inside a drive position hole. Elements are connected with cyanoacrylate unless otherwise noted.

The tubing axis of the systemDrive is backfilled with hydrophobic lubricant (petroleum jelly, Vaseline) to prevent CSF-borne macromolecules or tissue from infiltrating the drive axis and binding the wire insulation to the polymer tubing, as well as potentially decreasing the chances of infection. In contrast to most other microdrives that require the advancement of electrodes from the brain surface, the lubricant-filled tube allows for deeper electrode implantation because the electrodes are protected as they traverse the cannula before entering the brain. Electrodes are fully withdrawn into the drive axis before implantation to protect their tips. Therefore, a small air pocket at the bottom of the placement tube is maintained to prevent the oil from obfuscating the electrode tips. The pocket is made at the time of cleaning the electrode tips in acetone before electrodeposition.

The rectangular drive springs are designed to fit the width of individual 2.5 mm drive positions. Springs are individually cut from an array so that they are only used where needed, as demonstrated in Figure 4*B*. Each drive spring has a polyimide tube that is attached with epoxy (Loctite 5 Minute, Henkel) that bridges the arms of the spring head. The shuttle tube is rigidly attached to this bridge with cyanoacrylate.

The microdrive body is the nexus for the various drive elements and auxiliary components that make up the complete microdrive system. Shown in Figure 4*D* are three-axis and isometric views of the microdrive body. The microdrive body has a 2 × 7 array of drive positions that are designed to hold one-quarter inch 000-120 screws. The array is angled to direct the long flexible implants, which are ∼25–28 mm in total length, to cross over the midline (Fig. 4*C*), and to provide compact screwdriver access below the EIB. The large array of drive positions provides flexibility for where FDAs can be mounted along the rostrocaudal axis. The array is raised a few millimeters from the bottom of the microdrive body to provide space to maneuver the FDA into the guide cannulas with forceps. At the spring wedge, the drive springs are fixed to the microdrive body with epoxy. The wedge is angled to enable smooth arcs of the springs as they are pushed down and outward.

The EIB and recording amplifier sit on top of the microdrive body (Fig. 4*B*). The EIB is secured with 00-90 screw columns on the drive body. The EIB has 32 gold-plated vias and a 36-position Nano Strip connector (catalog #A79026-001, Omnetics Connector). The Intan recording amplifier is plugged into the Nano Strip connector and is positioned vertically on the EIB.

The microdrive and all its sensitive components are protected by a headmount during chronic, freely behaving recordings. The headmount (Fig. 4*E*) is comprised of two separate parts, the base and the cap. A key feature of the systemDrive is the large open center of the headmount base. The width nearly spans the outer cranial ridges, and the length extends a few millimeters past the anatomic landmarks lambda and bregma. This opening provides sufficient access to the cranium for easy positioning of FDAs and auxiliary physiologic sensors for both MSMR single-unit observations and multimodal recordings.

### Microdrive placement

The method for positioning microdrive implants is dependent on the surgical placement of a guide cannula. A craniotomy is made along the target trajectory, and the guide cannula is positioned with a surgical stylus so that its dorsal end will prevent the FDA from advancing further. In conjunction with this core technique, the full microdrive placement is performed with the following key steps: (1) expose and prepare the skull, and implant other electrodes and sensors; (2) stereotaxically place the guide cannulas, which ensure that the FDAs are each individually stereotaxically positioned; (3) place and fix the headmount base to the skull; and (4) implant the microdrive. Slide each FDA through its guide cannula and secure the microdrive body to the headmount base.

The procedure for these steps are expounded in the rest of this section. Specific information for craniotomies and implants used in the current report are placed alongside the relevant step.

Craniotomies for each implant are made (Table 2), and the skull is prepared with OptiBond UV-cured dental adhesive (Kerr). Next, any other sensors are placed. In our case, one-eighth inch 18-8 stainless steel 00-80 cortical screws (Table 2; McMaster-Carr) and hippocampal depth electrodes (Table 3; Shanmugasundaram and Gluckman, 2011) were implanted. Following this, guide cannulas are positioned with the STU (Table 3) based on inputs to Equation 1. The cannulas are then fixed to the skull with Ortho-Jet dental acrylic (Lang Dental).

**Table 2:** Chronic implant craniotomies

Targets/coordinates	AP	ML
Anterior screws	11	±3
Middle screws	7.5	±4
Left posterior screw	3	−4
Right posterior screw	2.5, 3	3, 4
Depth electrode pairs	5	±2.5, ±3
PPT	0.5, 1	±2
DR	1.5	−2.5
LDT	0.36, 0.48	2, −2.5
VLPO	8.76	1.5, 2
LC	−0.72	1.3

AP and ML coordinates [Intreraural (IA)-referenced, in mm] for chronic implant craniotomies. Each subject had all craniotomies for screw and depth electrodes, plus selected craniotomies for SWRS targets. Coordinates are from Paxinos and Watson (2007).

**Table 3:** Chronic implant target coordinates

Targets/coordinates	AP	ML	DV	Angle
Depth electrodes	5	±2.5, ±3	7.2–7.8 (−2.0 to −2.6 from cortex)	$0^{{}^{\circ}}$
PPT	0.5, 1	±2	3.6	$0^{{}^{\circ}}$
DR	1.5	0	3.6	$21.33^{{}^{\circ}}$
LDT	0.36, 0.48	±0.8	3.0	$13.65^{{}^{\circ}}$
VLPO	8.76	±1	0.75, 1	12.2°, 15.12°, 15.52°
LC	−0.72	1.3	3.2	$0^{{}^{\circ}}$

Anterior-posterior (AP), medial-lateral (ML), and dorsal-ventral (DV) target coordinates (IA-referenced, units of mm) with the absolute trajectory angles for each implant. Coordinates are from Paxinos and Watson (2007).

Intermediate to the microdrive placement, guide cannulas are plugged with petroleum jelly-filled polyimide tubes to prevent CSF and/or blood from fouling them via capillary action. These plugs are made from 6.5 mm to 7.0-mm long, 6.3 mil polyimide tubes with short 8.9 mil polyimide tube collars that stop them from being inserted too far into the guide cannulas. Following placement of the plugs, the headmount base is secured to the cranium with dental acrylic.

The final step is the microdrive placement. This occurs either during surgery or following 3–7 days of postoperative recovery. For postoperative microdrive placement, animals are anesthetized and gently secured in a stereotaxic frame.

Microdrive placements consist of the following three main parts: insertion of the placement tubes into the guide cannulas, lowering and securing of the microdrive body to the headmount base, and riveting of accessory sensor leads to the EIB. The microdrive body is attached to a 3D-printed placement tool that allows the structure to be maneuvered with a stereotaxic device (Fig. 5*C*). Once the petroleum jelly plugs (Fig. 5*D*) are removed from the guide cannulas, the microdrive is lowered and the placement tubes are inserted manually into the guide cannulas (Fig. 5*E*). Forceps (Dumont #5, Fine Science Tools) are customized with a small groove to grip the polyimide tubes without crushing them (Fig. 5*F*).

Each axis has slack to move the microdrive structure in the x–y plane without risking kinking or explanting once they are inserted into the guide cannulas. This is useful for getting placement tubes as close to their respective guide cannula as possible before manipulating them with the forceps. The insertion of the placement tubes generally starts with the most rostral implant, then moves to the next adjacent implant. Once each placement tube is engaged with its respective guide cannula (Fig. 5*G*), the structure is slowly lowered and the placement tubes are further inserted until all the body tubes meet the guide cannulas (Fig. 5*H*). Once each drive axis is fully seated, the microdrive body is secured to the headmount base and the auxiliary sensor leads are riveted to the EIB (Fig. 5*I*).

The total amount of time the surgery and microdrive placement takes will scale with the number of FDAs implanted. After initial craniotomies are made, each guide cannula placement takes ∼15–20 min. Time estimates for microdrive placement can be broken down approximately as follows: 30 min for animal setup and positioning of the Microdrive; 5 min to slide each tube into place; and 10 min to advance placement tubes through their guide cannulas. Any riveting for leads of additional sensors can take, in total, 15 min. Thus, once practiced, the placement of additional drive axes, which includes guide cannula placement and FDA positioning, would add ∼30 min/axis to the overall surgery.

### Histologic validation and chronic recording data

The requirements for successful use of the systemDrive are accuracy, repeatability, and stability, particularly as follows: (1) accurately target multiple structures with microelectrode bundles simultaneously; (2) repeat this with multiple subjects; and (3) reliably record single units in freely behaving animals once in the targets.

Here, we report on 13 subjects that were implanted and recorded during the time period of February 2 to December 13, 2017, matching a time period when we had mostly refined our implanting, handling, and recording protocols. Four additional animals implanted in that time period expired before chronic/continuous recording. One other animal was excluded because it expressed a preponderance of seizure-like high-voltage rhythmic spiking events, which are SOV dependent (Shaw, 2004; Pearce et al., 2014; Taylor et al., 2017). Through the course of recording from these animals, our technique improved and we increased the number of recording axes from three to five. The majority of recordings were acquired with an Intan differential amplifier; the first animal had intermittent referential recordings and another had referential recordings for 1 h. A comparison of differential to referential data showed that differential recordings were less susceptible to noise due to motion artifact or impedance mismatches between microwire electrodes and other different-sized electrodes used for lower-impedance recordings and reference.

Presently, the systemDrive has been used for multimodal and single-unit chronic recordings on multiple subjects with targeting of up to five simultaneous sleep–wake regulatory target populations using bundles of four or six electrodes. The SWRS targets include the pedunculopontine tegmental nucleus (PPT), laterodorsal tegmental nucleus (LDT), dorsal raphe (DR), ventrolateral pre-optic nucleus (VLPO), and locus coeruleus (LC). Implant sites were widely distributed spatially across rostrocaudal and mediolateral coordinate axes, and each SWRS target required a different FDA trajectory (Table 3). Driving distances Δ varied from 1 to 2 mm for the targets.

#### FDA targeting accuracy and recording success

**Figure 6.**
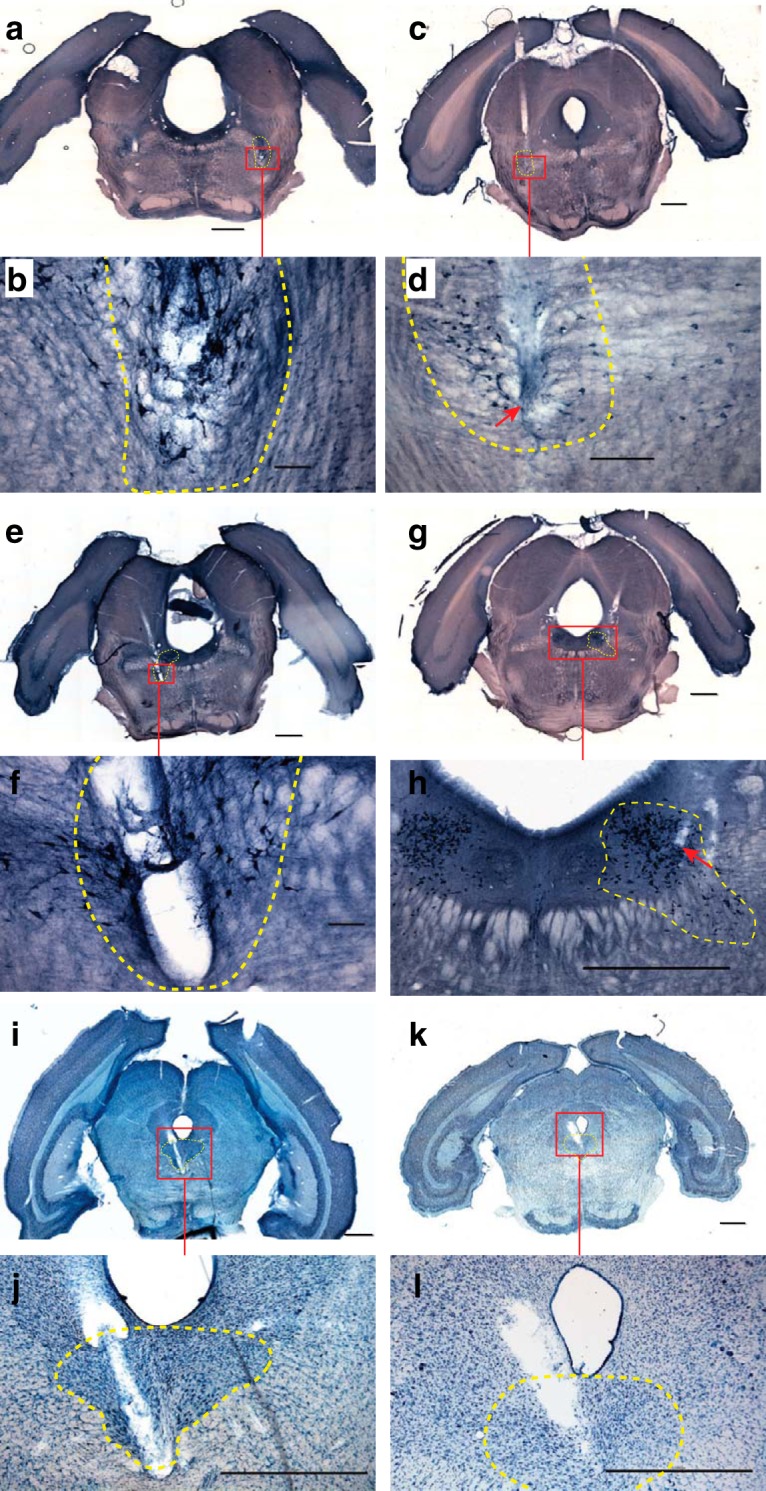
Targeting accuracy across multiple subjects. Coronal histologic sections taken from a total of four different chronically recorded subjects that demonstrate FDA targeting accuracy and repeatability from multiple brainstem areas. The target structures displayed include the following: bilateral PPT, bilateral LDT, and DR. Implant coordinates and angling for targets are in Table 3. The left column contains histology from three separate subjects, all of whom had other FDA sites. The right column contains histology from a single subject. ***a–d***, NADPH-stained coronal sections that show electrode tracks at bilateral PPT regions (targeting parameters for left column, right column in mm: *R* = 9.17, 9.28; α = 7.21, 7.41; Δ = 1). ***e*–*h***, NADPH-stained coronal sections that show electrode tracks at bilateral dorsal and ventral LDT regions (*R* = 9.20, 9.28; *α* = 7.16, 7.41; Δ = 2). ***i*–*l***, Nissl-stained coronal sections that show electrode tracks at the DR nucleus (*R* = 8.83, 9.28; α = 7.02, 7.41; Δ = 1, 1.25). The red boxes indicate approximate areas from whole-slice images, which are zoomed-in on subsequent images. The yellow dashed lines indicate the estimated boundary of target structures. The red arrows indicate the estimated end of electrode tracks. Scale bars: ***a***, ***c***, ***e***, ***g***, ***h***, ***i***, ***j***, ***k***, ***l*** (4× magnified images), 1000 µm; ***b***, ***f*** (10× magnified images), 100 µm; ***d*** (10× magnified images), 200 µm.

Typical targeting accuracy is shown across histologic sections from multiple subjects (Fig. 6). These sections illustrate electrode tracks from individual FDAs to their respective target brain regions. Cholinergic PPT and LDT neurons are visualized in NADPH-stained sections (Fig. 6*A–H*). The DR region appears in Nissl-stained sections underneath the cerebral aqueduct (Fig. 6*I–L*). The targeting of LDT and DR (Fig. 6*E–L*) required that FDA trajectories be at nonzero angles to avoid damaging sensitive fourth ventricle or cerebral aqueduct midline structures. The DV coordinates of PPT, LDT, and DR are relatively similar, so targeting parameters *R* and α did not vary much between targets.

Shown in Tables 4 and 5 are the success rates of chronic FDA targeting and recording from multiple (*n* = 13) subjects. In Table 4 each FDA target was categorized by whether the electrode tracks reached the target SWRS structure, and whether unit activity characteristic of cell groups within those structures were recorded. Session-by-session counts of well identified units per target are provided in Table 5 to demonstrate session-by-session variation in unit detection. These waveforms were stable for a minimum of 3 d during continuous recordings and changed in shape and amplitude as we advanced the electrodes. The activity of each neuron was analyzed as a function of transitions between different SOV values and time spent in each state. SOV-dependent neurons were validated based on whether they had significantly higher firing rates during different SOVs and were compared with previous reports on state-dependent neuronal activity both in terms of firing rate and waveform characteristics (Gervasoni et al., 2000; Datta and Siwek, 2002; Wu et al., 2004; Dahan et al., 2007; Sakai, 2011; Boucetta et al., 2014; Cho et al., 2017).

**Table 4:** Success rates of chronic FDA targeting: cumulative neurons per target

Animal	Recording duration (d)[no. of sessions]	PPT		LDT		DR	VLPO	LC
		Left	Right	Left	Right		Right	
1	42 [13]	NU	NU			**23 (47)**		
2	21 [5]	NU	NU			NU		
3	37 [12]	**21 (42)**	**13 (33)**			**17 (43)**		
4	57 [9]	**19 (29)**	*8 (29)*			**10 (18)**		
5	73 [12]	*1 (7)*	NU			*7 (18)*		
6	66 [10]	**9 (20)**			**4 (14)**	*10 (27)*		
7	43 [8]	**20 (24)**			**22 (29)**	**11 (32)**	*10 (26)*	
8	39 [7]		**4 (6)**	**12 (19)**		*2 (3)*	*4 (9)*	
9	54 [9]	Kinked			**28 (47)**	**31 (44)**	NU	
10	57 [9]	**6 (8)**			**21 (43)**	*3 (23)*	*5 (13)*	
11	34 [8]		**27 (42)**	Kinked		**9 (15)**	**6 (16)**	
12	29 [8]		**17 (25)**	**19 (30)**		NU	**5 (16)**	
13	39 [6]		**14 (30)**	**7 (16)**		Kinked	**3 (8)**	**0 (4)**
SOV-dependent unit count (total unit count)		159 (295)		113 (198)		123 (270)	33 (88)	0 (4)

Success rates of animals with simultaneous chronic recordings from multiple SWRS targets. The cells include information about which targets had FDAs as well as the number of well defined SOV-dependent and total units counted over the entire course of recordings for each axis and animal. The final row is the sum of SOV and total units over all animals in the cohort. Animals 1–6 had three FDAs, animals 7–12 had a total of four FDAs, and animal 13 had a total of five FDAs. Formatting of cells refer to the following conditions: recorded units with histologic validation that the electrodes hit the target (bold); recorded units with no histologic validation (italics); recorded activity but no units crossed the 7 SD threshold (NU). Some implanted FDAs got kinked, which prevented electrodes from driving. Sessions were continuous 24 h/7 d/week recording periods between electrode driving sessions. Recording sessions typically lasted between 5 and 7 d.

**Table 5:** Success rates of chronic FDA targeting: recorded neurons per session

Animal	PPT	LDT	DR	VLPO	LC
1			[6, 2, 3, 4, 2, 3, 3, 4, 8, 5, 1, 3, 3] (3.61 ± 1.85)		
2					
3	[4, 2, 5, 6, 6, 8, 11, 7, 9, 5, 4, 8] (6.25 ± 2.49)		[6, 2, 4, 6, 6, 5, 5, 3, 2, 1, 2, 1] (3.58 ± 1.97)		
4	[5, 6, 7, 5, 8, 7, 7, 6, 7] (6.44 ± 1.01)		[2, 4, 2, 2, 2, 1, 0, 2, 3] (2 ± 1.11)		
5	[0, 0, 2, 0, 0, 1, 0, 0, 0, 1, 1, 2] (0.58 ± 0.79)		[1, 2, 3, 2, 3, 2, 0, 1, 1, 0, 0, 3] (1.5 ± 1.16)		
6	[0, 0, 0, 0, 1, 3, 4, 4, 3, 5] (2 ± 2)	[0, 0, 0, 2, 2, 1, 3, 3, 1, 2] (1.4 ± 1.17)	[1, 3, 3, 2, 3, 3, 4, 3, 4, 1] (2.7 ± 1.05)		
7	[4, 3, 3, 2, 3, 4, 2, 3] (3 ± 0.76)	[5, 2, 3, 4, 5, 4, 2, 4] (3.62 ± 1.88)	[5, 5, 4, 3, 5, 4, 2, 4] (4 ± 1.07)	[6, 3, 3, 4, 4, 2, 1, 3] (3.25 ± 1.49)	
8	[0, 2, 2, 1, 0, 0, 1] (0.85 ± 0.9)	[0, 0, 6, 3, 4, 3, 3] (2.71 ± 2.14)	[0, 0, 1, 0, 1, 1, 0] (0.43 ± 0.53)	[3, 2, 3, 1, 0, 0, 0] (1.28 ± 1.38)	
9	Kinked	[6, 7, 5, 6, 5, 5, 4, 6, 3] (5.22 ± 1.2)	[1, 1, 4, 7, 6, 6, 8, 5, 6] (4.88 ± 2.47)	[0, 0, 0, 0, 0, 0, 0, 0, 0]	
10	[0, 0, 0, 0, 4, 0, 0, 3, 1] (0.88 ± 1.53)	[6, 5, 3, 4, 8, 3, 3, 7, 4] (4.77 ± 1.85)	[0, 2, 3, 0, 5, 3, 4, 2, 4] (2.56 ± 1.74)	[6, 1, 2, 0, 2, 1, 1, 0, 0] (1.44 ± 1.87)	
11	[1, 7, 8, 4, 6, 6, 3, 7] (5.25 ± 2.37)	Kinked	[5, 3, 0, 0, 0, 0, 3, 4] (1.87 ± 2)	[1, 2, 5, 3, 2, 3, 0, 0] (2 ± 1.69)	
12	[5, 2, 3, 3, 3, 3, 2, 4] (3.12 ± 0.99)	[5, 3, 3, 3, 5, 2, 5, 4] (3.75 ± 1.16)	[0, 0, 0, 0, 0, 0, 0, 0]	[5, 3, 2, 3, 0, 0, 2, 1] (2 ± 1.69)	
13	[6, 6, 5, 3, 5, 5] (5 ± 1.09)	[0, 4, 6, 3, 1, 2] (2.66 ± 2.16)	Kinked	[2, 3, 2, 1, 0, 0] (1.33 ± 1.21)	[2, 0, 0, 2, 0, 0] (0.67 ± 1.03)

Number of neurons recorded from each target per recording session, unless the axis was kinked or each session had zero neurons: [no. of neurons per session] (mean ± SD). Each recording session occurred after one or more axis was moved forward during a driving session. The units included were stable for at least three days during continuous recording periods.

For each subject, the recording period in days, the total number of recording sessions, and the total number of single units detected with a threshold of 7 SDs are presented in Tables 4 and 5. Recording sessions generally began when the electrodes were estimated to be in their target structures from drive distance and electrophysiology. Each successive recording session had at least one of the electrode bundles at a new ventral location within the targets.

#### Long-term recording and stability

The systemDrive enables stable long-term measurements from many brain regions using different modalities, such as single-unit and multiunit recordings from SWRS cell populations, and ECoG and hippocampal LFP. Unit waveforms are stable over continuous recording periods of days to weeks, and the overall microdrive structure is robust against mechanically induced artifacts caused by behaviors such as grooming and headmount scratching.

**Figure 7.**
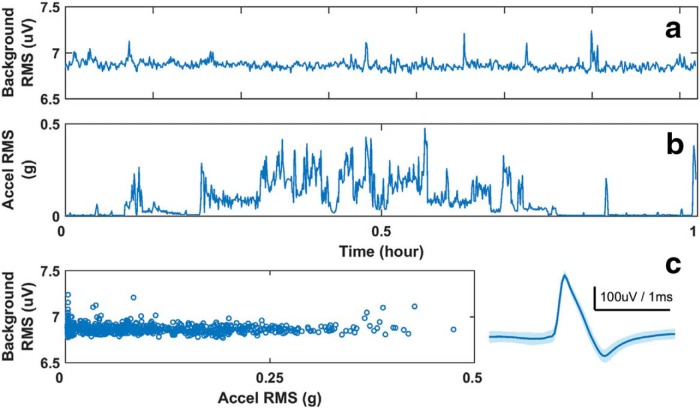
Chronic recording stability. ***a***, ***b***, For an hour-long block of data background, the RMS of the time series from on the microwire in dorsal raphe (***a***) and one accelerometer channel (***b***) are computed over 5 s long overlapping windows (overlap = 1 s). ***c***, The RMS background of the microwire channel remains flat with respect to the accelerometer activity during the hour. The maximal range of the background noise (∼7 µV) is much smaller than the amplitude of the action potentials recorded on that channel (***c***, right).

Shown in Figure 7 is the RMS of the background noise from a typical microwire pair in DR (Fig. 7*A*) and the RMS of accelerometer activity (Fig. 7*B*) over a 1 h recording period. From the accelerometer activity, the animal was moving for the better part of the hour, with large head motions between minutes 15 and 40. However, there was not a significant change in the microwire RMS background as the acceleration RMS fluctuates (Fig. 7*C*), as might be expected if there were large motion artifacts. The consistent low background noise level (∼7 µV) was negligible compared with the amplitude of the recorded action potential from that channel (Fig. 7*C*, right). The overall stability of the recordings means that unit detection is not disrupted during normal animal movements.

**Figure 8.**
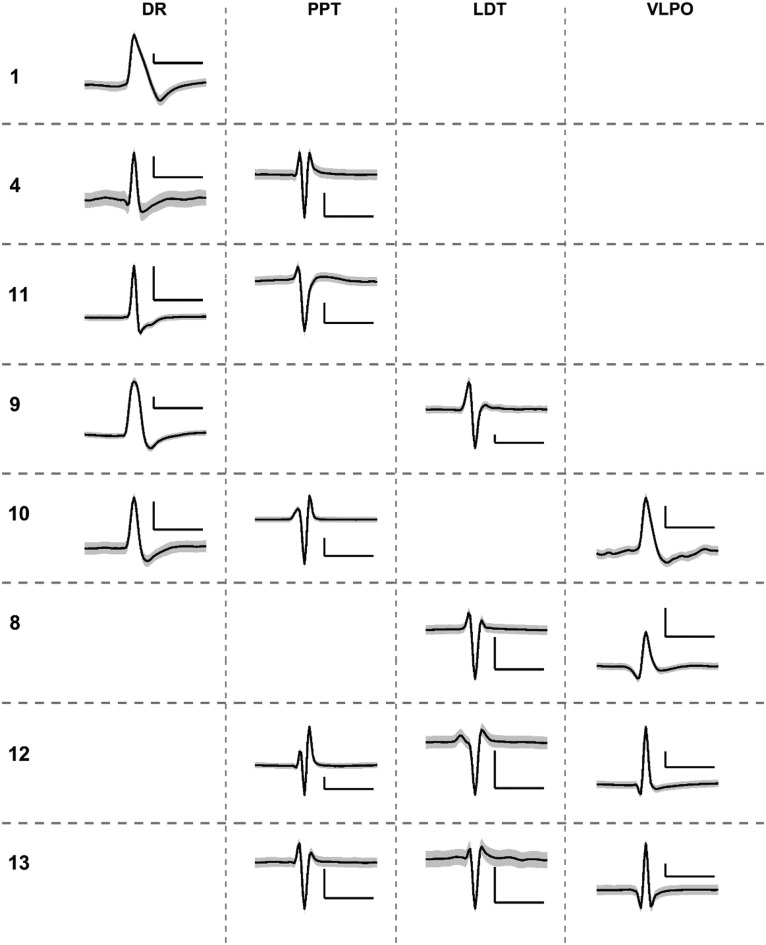
Simultaneously recorded single units. Example single unit action potentials simultaneously recorded from up to three target SWRS structures. Units across (*n* = 8) subjects were classified as state dependent by observing changes in firing rates during and outside of characteristic sleep–wake states. The subject numbers correspond to those in Table 4, but the order has been rearranged for the sake of grouping units from the same structures. The *y*-axis and *x*-axis of each scale bar represent 50 µV and 1 ms, respectively.

We successfully measured state-dependent neuronal activity in multiple SWRS cell populations (Fig. 8). The unit waveforms were continuously recorded over 4–7 d from a subset of animals presented in Tables 4 and 5. These units were simultaneously extracted from up to three SWRS targets, and are the first report of chronic measurements from more than two nonparallel SWRS structures in chronic freely behaving animals. We validated each waveform based on the histologic analysis and previous reports of the shape and amplitude characteristics of action potentials from each cell group (Gervasoni et al., 2000; Datta and Siwek, 2002; Dahan et al., 2007; Sakai, 2011).

**Figure 9.**
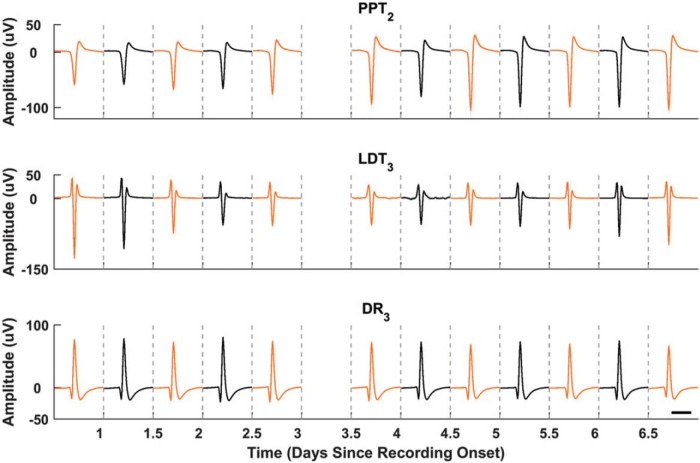
Chronic single-unit stability. Extracellular action potentials recorded simultaneously from PPT, LDT, and DR over a continuous 7 d recording session. Waveforms were averaged over 12 h periods to correspond with light (black traces) and dark (orange traces) cycles, which were separated because of the change in time-of-day dependence of the firing rates. The *x*-axis is days starting from onset of recordings in a particular recording session. The number subscripts for each region represent the group electrode channel on which each unit was recorded. Data missing from part of day 3 were the result of a computer acquisition failure. Scale: 1 ms.

Unit waveforms remained stable throughout the duration of recording periods with no deterioration of amplitude and signal-to-noise ratio (Fig. 9). Simultaneously recorded action potentials from PPT, LDT, and DR structures were recorded over 6 continuous days. These structures are active during different sleep–wake behaviors, and we separated waveform analyses into light and dark cycles to account for widely different firing rates. Nonetheless, the shape and amplitude of the average waveforms over the different cycles remained stable for the entirety of the recording session.

**Figure 10.**
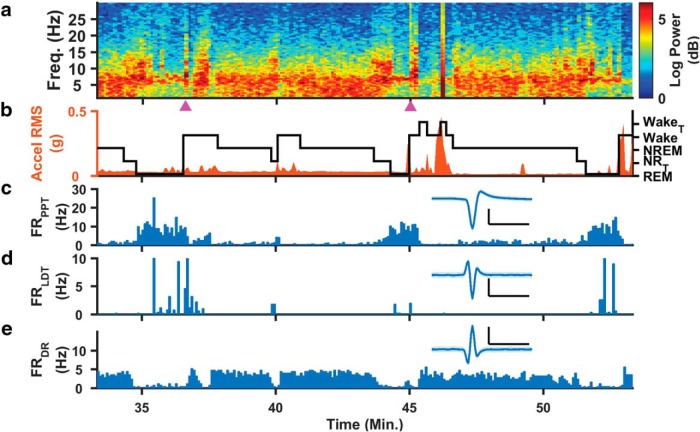
Multimodal neural circuit recordings. The Microdrive provides a suite of measurements including hippocampal LFPs, ECoG, and unit recordings from multiple brainstem cell groups. ***a–e***, From a typical daytime hour of continuous recording, we extracted the power spectral density (PSD) of one LFP channel (***a***), one channel of head acceleration (orange trace) with hypnogram overlaid (black trace; ***b***), and single-unit firing rates from three different SWRS structures (PPT, ***c***; LDT, ***d***; DR, ***e***), with the inset representing the hour-long average waveforms. Based on the PSD and accelerometer activity, we find that PPT and LDT neurons are REM-on while the particular neuron in DR is REM-off. Magenta triangles indicate periods of high-amplitude activity similar to what is reported in high-voltage rhythmic spike discharges (Pearce et al., 2014; Taylor et al., 2017; Shaw, 2004). Hypnogram legend: Exploratory wake with hippocampal theta rhythm (Wake_T_), Wake, NREM, REM, transitions between NREM and REM (NR_T_). Scale, 50 µV, 1 ms.

The long-term stability of our measurements allows for high-resolution investigation of sleep–wake regulation. Shown in Figure 10 are hippocampal spectral (Fig. 10*A*) and accelerometer band (Fig. 10*B*) power, and a hypnogram (Fig. 10*B*), along with the firing rates of three different rapid eye movement (REM)-on and REM-off populations (Fig. 10*C–E*) during an hour of measurements from a single animal. The hypnogram was generated from a combination of hippocampal LFP and ECoG signals, and accelerometer activity (Sunderam et al., 2007; Sedigh-Sarvestani et al., 2014). During periods of wake activity, PPT and LDT fire less in REM-on populations, while REM-off DR neurons fire at a consistent 3–5 Hz rate. When the spectral EEG power increased in the 6–8 Hz range and the animal transitions into REM sleep, however, PPT and LDT increased their activity and DR had a markedly lower firing rate. These results are consistent with those of previous reports in head-fixed or acute single-structure studies (Datta and Siwek, 2002; Wu et al., 2004; Sakai, 2011; Boucetta et al., 2014; Cho et al., 2017).

## Discussion

The systemDrive is the first microdrive designed specifically for large-scale systems neuroscience. The flexible drive axis system makes possible independent positioning of trajectory angle and depth for many individual implants. This innovation helps to overcome the challenges of studying widely distributed neural circuits at high spatial and temporal resolutions using movable microwire electrode bundles. In the current study, the systemDrive was used to record unit activity from up to five noncoplanar, noncollinear targets of the SWRS network (PPT, LDT, DR, VLPO, and LC), along with ECoG and LFP recordings of cortex and hippocampus in freely behaving rats for months at a time.

Results from histologic sections showed accurate and repeatable FDA targeting of brainstem structures across multiple surgeries. Furthermore, the systemDrive proved to be very stable for chronic freely behaving recordings. Units in SWRS cell groups were recorded for continuous multiday periods without interruption from mechanical artifacts, and baseline noise remained low during times of increased animal movement. This stability enabled investigation into sleep–wake regulation with simultaneous activity from three or more noncollinear SWRS structures, plus EEG and accelerometer activity, for the first time in freely behaving rats.

### Comparison with other multisite, multiregion microdrives

The existing group of single-body multisite, multiregion microdrives suitable for small animal research derives the structure from the original hyperdrive design (Gothard et al., 1996), wherein the bodies are a cone shape and the electrode bundles funnel toward the base of the cone and out one or more separated exit cannulas. The majority of these drives have exit cannulas that are parallel and nonindependent (Lansink et al., 2007; Yamamoto and Wilson, 2008; Liang et al., 2017), such that all electrodes are placed and driven along parallel trajectories. Therefore, to achieve an angled electrode trajectory, the entire microdrive body must be must be tilted. Additionally, the multisite and multiregion capabilities of these designs are limited by the maximum number and separation of electrode sites that fit within the base of the drive. Thus, the customization of targets between experiments might require reworking the body design.

The “Quad-Drive” (Bos et al., 2017), which is derived from the original Split-Drive (Lansink et al., 2007), has a hyperdrive-like body and multiple exit cannulas, but differs from other designs in that its exit cannulas can be individually angled to direct electrode bundles along nonparallel driving axes. The orientation of exit cannulas is customizable, so there is a wider range of targeting options compared with similar MSMR microdrives. However, the Quad-Drive is also limited by its single exit hole at the base, so entry point and target region selection are not truly arbitrary, and exit cannulas must be angled in nonconflicting ways with each another since they are still closely grouped. As with other designs, its exit cannulas do not start from independent positions, and it might be questioned whether the cumulative trajectory targeting resolution achieved can be considered stereotaxically precise, as accuracy depends not only on the ability to place the drive body with respect to head fiducials, but also requires extremely high precision in bending of the output cannulas (see electrode targeting spread in Bos et al., 2017, their Supplementary Fig. 1D). Furthermore, possible benefits that could be gained from having the cannulas penetrate cortex are countered by the additional damage that would be caused by the forked rigid geometry of the cannulas, so in practice we understand that electrodes must be driven from the cortical surface.

The systemDrive was developed with the design consequences of other MSMR microdrives in mind. The main objective was to implement independent and arbitrary electrode axis angling, such that targeting would have stereotaxic precision at any depth and drive axes could be oriented to avoid sensitive anatomical structures. This was achieved by deviating from the legacy Hyperdrive body design in favor of an open-platform structure. Entry points and electrode trajectories of the systemDrive can be arbitrarily positioned with much wider separation and control. We believe that this provides a significant flexibility and advantage to our approach compared with other designs.

### Electrode motion in the flexible drive axis

When compared to more traditional microdrive designs, the extra travel distance and potential additional friction along the greater length and curvature of the FDA did not cause a noticeable problem with electrode “binding” nor did it interfere with advancing the electrodes to our targets in macrosteps (120–240 µm) or microsteps (35–70 µm) . In the same fashion as other studies (Lansink et al., 2007; Battaglia et al., 2009; Voigts et al., 2013; Bos et al., 2017), we estimated electrode tip location by counting the number of screw turns relative to our starting location away from the target (≥1000 µm), and by observing electrodes moving from neuron to neuron across drive sessions. This was subsequently verified against histologic slices that showed electrodes reliably traversing their targets. Occurrences of underdriving were rare and seemed to be in line with anecdotes from reports of other multisite drives (Lansink et al., 2007; Bos et al., 2017).

### Cost, scalability, and design improvements

The systemDrive is intended to be inexpensive to manufacture, relatively easy to construct, and scalable. The material cost of 3D-printed parts, polyimide tubing, and nichrome microwire is approximately $40 per drive. The most expensive components are the EIB and Omnetics connector, although commercial EIBs such as the Neuralynx EIB-36-PTB can also be used. Each drive takes ∼1–2 d to construct. The drive, with all its components, weighs ∼14 g, which is well under the head-weight carrying capacity of rats (Fee and Leonardo, 2001).

Currently, the EIB supports up to 32 electrode connections. The systemDrive could, however, support up to 128-channel unipolar recording channels with a few minor changes to the EIB, the addition of another Intan amplifier, and a modification to the headcap that expands its opening. An additional improvement to the systemDrive would be to reduce its overall height, which is currently limited by the amplifier and the EIB. The height of the construct could be reduced with the development of a daughter board that had a built-in Intan RHD2000 amplifier chip and a three-axis accelerometer, which plugged into a connector on the EIB.

In its current form, the systemDrive is optimized for rats. The microdrive body and headmount base have maximal open areas atop the rat cranium, which provide the ability to target trajectories entering from almost anywhere within the outer cranial ridge. The major design elements (open frame, FDA, surgical approach) could be adapted for mice or other animals through appropriate scaling of the frame, tube lengths, and number of insertion sites.

### Optical fiber and single-axis multitetrode integration

Many microdrives now offer integration of optical fibers for simultaneous electrophysiological recording and optogenetic control of target cell populations (VersaDrive-8 Optical, Neuralynx; Anikeeva et al., 2012; Voigts et al., 2013; Freedman et al., 2016; Liang et al., 2017). However, these designs are limited to single-drive axis targeting, which leaves the potential for large-scale circuit optogenetic interrogations using microdrives untapped. The systemDrive is well suited for multisite, multiregion targeting across the entire brain. The addition of small flexible fiber optics to electrode bundles, or integrated electrode-optic polymer fibers (Park et al., 2017), would open access to the control and observation of widely separated circuit connections in a chronic freely behaving context.

Another feature of other, high-density, microdrives is the ability to send multiple tetrodes down a single axis (Halo Microdrive, Neuralynx; Gothard et al., 1996; Yamamoto and Wilson, 2008; Voigts et al., 2013; Brunetti et al., 2014; Liang et al., 2017) to perform highly parallel recordings of many sites within a local region or structure. Currently, the systemDrive is only able to send single tetrodes or hexatrodes down individual FDAs. Multiple independent tetrodes could, however, be combined into a single drive axis if they all converged into the same placement tube from separate body tubes. This could be done by using a small flexible adapter that fixed multiple body tubes at its top and funneled tetrodes to a single exit point.

### The systemDrive: a solution for systems neuroscience

One goal of systems neuroscience is to elucidate the neurophysiological activity between specific cell groups during specific behaviors, and to understand how those smaller-scale interactions dynamically couple to global brain states. The multisite, multiregion recording capabilities of the systemDrive provide the ability to do this by enabling simultaneous recordings from many separate structures over a large dorsal-ventral, medial-lateral, and anterior-posterior range on a single subject. This ability could be useful in studying the electrophysiological dynamics of newly discovered learning (Wagatsuma et al., 2018) and memory consolidation (Kitamura et al., 2017) circuits that have widely distributed cell populations across the hippocampus, cortex, and brainstem. The MSMR capabilities could also be used for the chronic free-behavior investigation of bilateral thalamostriatal circuits previous only studied in head-fixed animals (Watson et al., 2015).

In addition to its MSMR features, the systemDrive supports multimodal recordings by providing full access to the cranium for other auxiliary implants. A multimodal setup like the one used in the current study would be ideal for the chronic observation of thalamocortical networks thought to be responsible for absence seizures in models of epilepsy (Pearce et al., 2014; Taylor et al., 2017) and the mechanisms relating spike-wave discharges in ictal-like thalamocortical dynamics and sleep (Shaw, 2004; Dewolfe et al., 2013). Other sensor modalities, such as cardiac and respiratory electrodes, and oxygen sensors, could be added to study the relationship between seizures and sudden unexpected death from epilepsy (Ssentongo et al., 2017; Bahari et al., 2018).

In conclusion, the systemDrive is a novel neurophysiology tool designed to expand the current capabilities of systems neuroscience. The core findings of this report demonstrated the ability of our microdrive to reliably record stable and relevant unit activity in multiple brainstem structures, with the addition of other electrophysiolgical modalities, across a cohort of 13 animals. The features of the systemDrive provide the flexibility to target many widely separated sites anywhere in brain with independently positionable and movable electrode bundles, thus enabling researchers to investigate distributed neural circuits chronically with high spatial and temporal resolution at a low cost. We believe this work is an important step in accelerating the ability of the neuroscience community to elucidate the dynamics of many interconnected brain structures and nuclei, and how such dynamics mediate large-scale behaviors.

## Synthesis

Reviewing Editor: Thomas McHugh, RIKEN Center for Brain Science Decisions are customarily a result of the Reviewing Editor and the peer reviewers coming together and discussing their recommendations until a consensus is reached. When revisions are invited, a fact-based synthesis statement explaining their decision and outlining what is needed to prepare a revision will be listed below. The following reviewer(s) agreed to reveal their identity: Umberto Olcese.

Thank you for your submission. After consulting with the referees we have reached a consensus that current manuscript can be reconsidered for publication at eNeuro following revision.

Both referees found the paper interesting, summarizing the work as follow: “This manuscript describes the development of a novel microdrive system to reliably record from multiple small and deep brain areas that are crucial for sleep-wake regulation (SWR) in rats. These brain areas locate in the middle of the brain where standard methods may cause critical damages along the way to the targets. The author's non-parallel, multiple angles approach using flexible drive axis (FDA) may be one of the novel approaches to solve this kind of issue. The authors demonstrated that they successfully recorded from ventrolateral pre-optic nucleus (VLPO), laterodorsal tegmental nucleus (LDT), pedunculopontine tegmental nucleus (PPT), dorsal raphe (DR), and locus coeruleus (LC) simultaneously across multiple days at decent recording stability.”

Reviewer 1 added “Although I am overall intrigued by the novel approach, the current form lacks some of the crucial points in order for other scientists to consider incorporating this system” and reviewer 2 commented “The manuscript provides a novel approach to perform multi-area tetrode recordings. The authors' claims are in my opinion a bit exaggerated, as methods to perform recordings of multiple, arbitrarily located areas already exist (see my comments to the authors for details). Anyway, the approach developed to connect multiple cannulas to a single tetrode drive is innovative.”

Discussing the reviews lead to the recommendation the authors address the following 3 main points:

1) One concern is the repeatability of electrodes tip movements, especially in the brain tissue. Many scientists look for well isolated single units by repeatedly displace the electrode tips up and/or down at very fine steps. The current form does not evaluate how smoothly the electrode tips move. One concern is the bent flexible guide/body tubes (FDA) may substantially increase friction within the concentric tubing so that the responsiveness of electrode tips to smaller displacements may be affected. This point should be addressed in the revised version.

2) The idea of modular design is great but also there will be many critical steps during surgery. The authors claim that the current design can hold up to 14 independently moveable sites. However, the demonstration in the current form only uses 4-5 sites plus several fixed depth electrodes and total duration of such surgery is not well described. The authors should demonstrate how long it would take to implant more than 10 (or close to maximum 14) non-parallel FDAs regardless of the region of interests of the SWR study. It is also useful to share critical points during microdrive fabrication, surgery and adjusting procedures.

3) Contrary to the authors' claim, alternative methods able to achieve the same objective in rodents already exist (see e.g. Vinck et al., Frontiers in Systems Neuroscience, 2016; Bos et al., Nature Communications, 2017; Olcese et al., J Neurosci, 2016; Olcese et al., BiorXiv, 2018). The authors should acknowledge this (and revise the significant statement) and discuss the differences between their approach and alternative ones. For instance, existing approaches allow to record from 4 regions, with arbitrary (non-parallel) orientations of tetrode bundles (multiple tetrode reaching the same area). What are the advantages and disadvantages of the proposed method? The authors should also discuss more in depth the advantages and disadvantages with respect to competing technologies such as fixed silicon probes (Okun et al, Plos ONE, 2016), and in particular Neuropixel probes (Jun et al., Nature, 2017).

In addition, the referees raised several minor points to address in order to improve presentation and clarity:

1) In Figure 1, scale bar or more specifically, depth information for each recording site is needed. Authors should emphasize how difficult it is to hit all of the deep brain areas all at once in the main text as well.

2) In Figure 2, again the scale bars are needed. I understand that the authors had to exaggerate the schematic, especially on the panel (a). However, the panel (b) will provide negative impression of the potential damage to the upper half of the brain. The authors need to reconsider the drawing.

3) In Figure 2, it might be useful to show the real picture of back side of the post mortem skull to show how non-parallel FDAs are inserted in the brain.

4) Figure 3 is extremely confusing at the beginning as the main text does not clearly explain a) why there are two kinds of tubing and b) what they are for. I could finally figure out how it works when I reached Figure 5. Authors should reconsider the explanation of FDA and STU in the main text. It might also help if you can provide multimedia files (actual video and/or animation of 3D CAD design) as supplement. Also, in Figure 2 and 3, it would be good to have information about the diameter of tubes.

5) Figure 4 can be better and easier to understand how it works by creating an explode view of the drive system including the Stereotaxic Targeting Devices shown in Figure 3.

6) Figure 5 should be bigger. Otherwise, it is very difficult to follow the procedures.

7) In Figure 6, it would be helpful to highlight each target area with dashed-lines, especially on the magnified panels. Readers will have no idea where the planned targets are in the figure.

8) In Figure 7, unit of Accel RMS should be converted to (G) not as a sensor output (mV).

9) In Figure 9, why D3 data is missing? It would be more straight forward to show time (x-axis in minutes or hours) vs spike amplitudes (y-axis in uV) plots to assess unit stability.

10) The Visual Abstract may include some data panels (ex, Fig.10) to emphasize how difficult it is to record multiple SWR areas simultaneously in rats.

11) It is important to compare the proposed method to other approaches for multi-area recordings, in which only tetrodes (and not guide tubes) are inserted in brain tissue. Related to this, the authors should clearly discuss to what extent superficial brain structures (cortex) can or cannot be recorded with the proposed drive.

12)A photograph of a rat with the drive would help understand the end result.

13) The results presented in Table 4 need to be better substantiated by a detailed description of the methods used to determine the results: 1) How was a neuron determined to be SOV-dependent? 2) How many neurons per sessions were recorded? Was there a variation in neurons/session over the course of recording periods?
